# Soluble urokinase plasminogen activator receptor and cardiotoxicity in doxorubicin-treated breast cancer patients: a prospective exploratory study

**DOI:** 10.1186/s40959-023-00191-0

**Published:** 2024-01-15

**Authors:** Jian Chu, Lillian Tung, Issam Atallah, Changli Wei, Melody Cobleigh, Ruta Rao, Steven B. Feinstein, Lydia Usha, Kathrin Banach, Jochen Reiser, Tochukwu M. Okwuosa

**Affiliations:** 1 Department of Internal Medicine, Rush University Medical Center, Chicago, IL USA; 2 Department of Internal Medicine, Division of Cardiology, Saint Louis University Hospital, St. Louis, MO USA; 3 Department of Internal Medicine, Section of Hematology/Oncology, Rush University Medical Center, Chicago, IL USA; 4 Department of Internal Medicine, Division of Cardiology, Rush University Medical Center, 1717 West Congress Parkway | Kellogg Bldg, Suite 328, Chicago, IL 60612 USA

**Keywords:** Soluble urokinase Plasminogen Activator Receptor (suPAR), Biomarker, Global longitudinal strain (GLS), Echocardiography, B-type natriuretic peptide (BNP), Troponin, Breast cancer, Doxorubicin, Cardiotoxicity

## Abstract

**Background:**

Soluble urokinase plasminogen activator receptor is an inflammatory biomarker that may prognosticate cardiovascular outcomes. We sought to determine the associations between soluble urokinase plasminogen activator receptor and established markers of cardiotoxicity in breast cancer patients receiving doxorubicin.

**Methods:**

We conducted a prospective cohort study of women with newly diagnosed breast cancer receiving standard-dose doxorubicin (240 mg/m^2^) at Rush University Medical Center and Rush Oak Park Hospital (Chicago, IL) between January 2017 and May 2019. Left ventricular ejection fraction, global longitudinal strain, and cardiac biomarkers (N-terminal prohormone B-type natriuretic peptide, troponin-I, and high-sensitivity C-reactive protein) were measured at baseline and at intervals up to 12-month follow-up after end of treatment. The associations between soluble urokinase plasminogen activator receptor and these endpoints were evaluated using multivariable mixed effects linear regression.

**Results:**

Our study included 37 women (mean age 47.0 ± 9.3 years, 60% white) with a median baseline soluble urokinase plasminogen activator receptor level of 2.83 ng/dL. No participant developed cardiomyopathy based on serial echocardiography by one-year follow-up. The median percent change in left ventricular strain was -4.3% at 6-month follow-up and absolute changes in cardiac biomarkers were clinically insignificant. There were no significant associations between soluble urokinase plasminogen activator receptor and these markers of cardiotoxicity (all *p* > 0.05).

**Conclusions:**

In this breast cancer cohort, doxorubicin treatment was associated with a very low risk for cardiotoxicity. Across this narrow range of clinical endpoints, soluble urokinase plasminogen activator receptor was not associated with markers of subclinical cardiotoxicity. Further studies are needed to clarify the prognostic utility of soluble urokinase plasminogen activator receptor in doxorubicin-associated cardiomyopathy and should include a larger cohort of leukemia and lymphoma patients who receive higher doses of doxorubicin.

**Supplementary Information:**

The online version contains supplementary material available at 10.1186/s40959-023-00191-0.

## Background

Cardiovascular disease (CVD) is a long-term sequela of cancer treatment and is the leading cause of mortality in breast cancer survivors [1–4]. Survivors experience higher incidence of congestive heart failure, atherosclerotic cardiovascular disease, and stroke compared with the general population.

The anthracycline doxorubicin (DOX) is the backbone of standard chemotherapy for breast cancers, lymphomas, and leukemias; but is associated with dose-dependent and irreversible cardiomyopathy and heart failure [5, 6]. Doxorubicin-induced cardiotoxicity has been attributed to multiple molecular pathways that ultimately lead to apoptosis of cardiomyocytes, which include dysregulation of intracellular calcium homeostasis, formation of reactive oxygen species, and disrupted autophagy [7]. Insights into these pathways offer promising avenues for prevention and treatment of DOX-induced cardiotoxicity [8].

At many cancer centers, patients are monitored for cardiotoxicity with serial echocardiographic assessment of left ventricular ejection fraction (LVEF). Unfortunately, the assessment of LVEF has poor sensitivity as a reduction in LVEF may signify late-stage, irreversible damage to cardiac tissue. Other surveillance markers such as global longitudinal strain (GLS) imaging and cardiac biomarkers – including N-terminal B-type natriuretic peptide (NT-proBNP), troponin I (TnI), and high-sensitivity C-reactive protein (hs-CRP) – provide earlier detection of subclinical cardiotoxicity [9–17].

Soluble urokinase plasminogen activator (suPAR) is an inflammatory biomarker that robustly predicts adverse cardiovascular events and mortality in patients with cardiovascular disease [18–20]. Furthermore, suPAR is shown to have prognostic utility among cancer patients, particularly breast cancer [21–24]. However, the prognostic utility of suPAR in DOX-induced cardiotoxicity has not been studied. We sought to evaluate the role of suPAR as an inexpensive and readily available predictor and/or marker of early or late subclinical cardiotoxicity in breast cancer patients receiving standard DOX chemotherapy.

## Methods

### Design and overview

This was a prospective, cohort study that recruited breast cancer patients between 18 and 64 years of age who underwent standard-dose DOX-based chemotherapy at Rush University Medical Center and Rush Oak Park Hospital (Chicago, Illinois) between January 2017 and May 2019. We excluded patients who were > 65 years of age as well as those with prior breast cancer treatment, advanced metastatic disease, HER2 positivity, reduced LVEF, or history of atherosclerotic cardiovascular disease (including angina, myocardial infarction, need for coronary artery bypass graft [CABG] or percutaneous coronary intervention [PCI], and end-stage renal disease [ESRD]). The study was approved by the Institutional Review Board at Rush University Medical Center (IRB #16022426) and all study participants gave written informed consent at the time of enrollment. A total of 42 patients were enrolled in the study and 5 withdrew their participation prior to study completion.

### Standard chemotherapy regimen

Patients underwent adjuvant chemotherapy with standard DOX and cyclophosphamide-based (AC) therapy for breast cancer with a dose-dense schedule of AC every two weeks for a total of four cycles that was followed by paclitaxel. Most women also received external beam radiation therapy during the one-year follow-up period.

### Study population and laboratory analyses

**Fig. 1:**
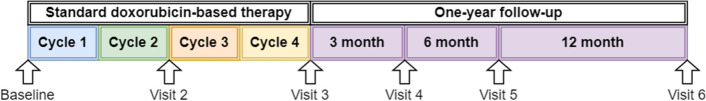
Serum biomarkers were measured at baseline, after each 2 cycles of chemotherapy, and at 3-month, 6-month, and 12-month follow-up (empty arrows)

We obtained baseline patient characteristics through chart review, which included traditional ASCVD risk factors (history of diabetes, current smoking status, hypertension, body mass index, dyslipidemia, family history of premature myocardial infarction) and current cardiac medications (aspirin, ACE-inhibitor/ARBs, beta-blockers, statins) at the time of their first chemotherapy cycle. We also obtained data on breast cancer characteristics, such as laterality, stage, grade, radiation history, surgical history, and adjuvant therapies. Blood draws were completed to obtain serum laboratory data that included suPAR, pro-BNP, troponin-I, and hs-CRP at baseline prior to chemotherapy; at each 4-week period during treatment; and at 3-month, 6-month, and 12-month follow-up (Fig. 1). Serum samples were stored at -70-80^o^ C and measured through an ELISA-based assay (suPARnostic®, ViroGates A/S, Birkeroed, Denmark). Troponin-I, pro-BNP, and hs-CRP were measured according to previously published methods [25–27].

### Echocardiography

Patients underwent serial echocardiograms with automated post hoc measurements of left ventricular GLS at baseline and at each visit. Two-dimensional images were obtained in standard 2-, 3-, and 4-chamber apical left ventricular views using a Philips IE-33 and myocardial strain was evaluated by speckle tracking analysis in these 3 apical views. Left ventricular GLS was calculated as an average of these measurements based on previously published methods [28]. Cardiomyopathy was defined based on European Society of Cardiology consensus guidelines as a reduction in LVEF by > 10% to a level < 53% and subclinical LV dysfunction was defined as a relative reduction in GLS by > 15% [12].

### Statistical analysis

Baseline patient demographics, cardiac risk factors, and breast cancer characteristics were described as mean (± standard deviation) for continuous variables and count (%) for categorical variables. The absolute and relative changes for each variable were reported at visit 3 (after chemotherapy) and at visit 5 (at 6-month follow-up). The primary endpoint was left ventricular GLS; while NT-proBNP, TnI, and hs-CRP were secondary endpoints. Multivariable mixed effects linear regression modeling was used to evaluate associations between baseline/serial serum suPAR measurements and these endpoints to account for correlation of measurements within individuals. This was specified as a two-level random intercept that was fit via maximum likelihood. The model adjusted for baseline demographics (age, race/ethnicity), ASCVD risk factors (history of hypertension, dyslipidemia, diabetes, BMI, current smoker, or family history), and cardiac medications (use of aspirin, statin, beta-blocker, or ACE-i/ARBs) [29]. Although mixed effect modeling was able to utilize all available data, the effect of missing observations (< 10% of measurements) were evaluated using multiple imputations in sensitivity analyses. A two-tailed *p*-value of < 0.05 was considered statistically significant for all analyses. Statistical analysis was performed using Stata 15.1 (StataCorp, College Station, TX).

## Results

### Patient population

Our study cohort comprised of 37 women after accounting for those who withdrew their participation (*n* = 5) (Supplementary Table 1). The women in our cohort were 47.0 ± 9.3 years of age and 60% were White (Table 1). Up to 35% of women had hypertension, dyslipidemia, and diabetes mellitus at baseline and up to 22% were on cardioprotective medications including ACE-inhibitors, ARBs, or beta-blockers. No woman had baseline chronic kidney disease. Sixty percent of women had left-sided breast cancer. Most women had stage II disease (70%) and hormone receptor-positive breast cancer (75%). Additionally, 86% of women underwent adjuvant radiotherapy within the one-year follow-up period.

**Table 1:** Baseline demographics, cardiovascular disease risk factors, cardiac medication use, and breast cancer characteristics

**Variables**		*N* = 37
**Age (yrs)**		47.0 ± 9.3
**White**		22 (60%)
**ASCVD Risk Factors**	**Hypertension**	13 (35%)
	**Dyslipidemia**	11 (30%)
	**Diabetes**	8 (22%)
	**Family History**	6 (16%)
	**Current Smoker**	3 (8%)
	**Body Mass Index (kg)**	29.6 ± 6.1
	**Chronic Kidney Disease**	0 (0%)
**Cardiac Medications**	**ACE-i/ARB**	8 (22%)
	**Beta-blocker**	6 (16%)
	**Statin**	5 (14%)
	**Aspirin**	2 (5%)
**Breast Cancer Characteristics**	**Left-sided**	22 (59%)
	**Stage I**	2 (5%)
	**Stage II**	26 (70%)
	**Stage III**	9 (24%)
	**ER + **	28 (75%)
	**PR + **	17 (50%)
**Treatment Characteristics**	**DOX dose (mg/m**^**2**^**)**	240.0 ± 0.0
	**Cyclophosphamide Use**	37 (100%)
	**Paclitaxel Use**	37 (100%)
	**Adjuvant radiation**	31 (86%)
**Baseline Cardiac Markers**	**GLS**	-20.2 ± 2.3%
	**suPAR (ng/dL)**	2.83 (1.31, 3.68)
	**NT-proBNP (pg/mL)**	31.9 (15.3, 90.7)
	**TnI (ng/dL)**	0.01 (0.01, 0.01)
	**hs-CRP (mg/L)**	3.6 (1.7, 10.1)

*Abbreviations*: *ACE-i* Angiotensin-converting enzyme inhibitor, *ARB* Angiotensin receptor blocker, *DOX* Doxorubicin, *ER* Estrogen receptor, *hs-CRP* High-sensitivity C-reactive protein, *NT-proBNP* N-terminal pro B-type natriuretic peptide, *PR* Progesterone receptor, *suPAR* Soluble urokinase plasminogen activator receptor, *TnI* Conventional troponin-I

Baseline suPAR was normal at 2.83 (1.31, 3.68) ng/dL. Additionally, baseline left ventricular GLS (-20.2 ± 2.3%) and baseline cardiac biomarkers including NT-proBNP (31.9 pg/mL; IQR: 15.3, 90.7), TnI (0.01 ng/dL; IQR: 0.01, 0.01), and hs-CRP (3.6 mg/L; IQR: 1.7, 10.1) were within their normal reference ranges.

### Associations between suPAR and cardiotoxicity endpoints

**Fig. 2:**
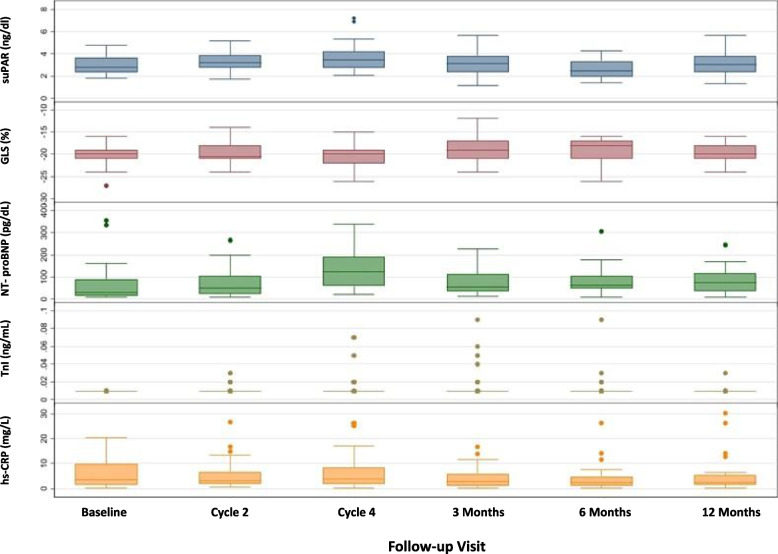
Changes in suPAR, GLS, NT-proBNP, TnI, and hs-CRP during study follow-up

No participants had developed clinically significant cardiomyopathy based on changes in LVEF by one-year follow-up. The mean change in suPAR at the end of the follow-up period was + 1.1%. The median relative percent change in GLS as the primary endpoint at 6-months of follow-up was negligible at -4.3%. Similarly, the median absolute changes in NT-proBNP (+ 18.9 pg/mL), TnI (0.00 ng/dL), and hs-CRP (-2.8 mg/L) at 6-month follow-up from baseline were clinically negligible (Table 2; Fig. 2). We did not report the changes at one-year follow-up as > 25% of participants failed to follow-up at that final visit.

**Table 2:** Absolute and relative percent changes in primary and secondary endpoints relative to baseline over follow-up

	**Visit 3 (after chemo)**	**Visit 5 (6-month follow-up)**
GLS		
Absolute change (%)	0 (-2, 1)	1 (-1, 2)
Relative change (%)	0.0 (-6.3, 10.5)	-4.3 (-10.0, 4.5)
NT-proBNP		
Absolute change (pg/mL)	66.9 (30.4, 138.5)	18.9 (-5.0, 42.1)
Relative change (%)	217 (86, 551)	67 (-5, 221)
TnI		
Absolute change (ng/dL)	0.00 (0.00, 0.00)	0.00 (0.00, 0.00)
Relative change (%)	0 (0, 0)	0 (0, 0)
hs-CRP		
Absolute change (mg/L)	-0.12 (-4.01, 1.16)	-2.75 (-6.06, 0.17)
Relative change (%)	-8.2 (-52.9, 66.8)	-53.9 (-74.2, 21.9)

Primary endpoint: GLS

Secondary endpoints: NT-proBNP, TnI, and hs-CRP

*Abbreviations*: *GLS* Left ventricular global longitudinal strain, *hs-CRP* High-sensitivity C-reactive protein, *NT-proBNP* N-terminal pro B-type natriuretic peptide, *suPAR* Soluble urokinase plasminogen activator receptor, *TnI* Conventional troponin-I

**Fig. 3:**
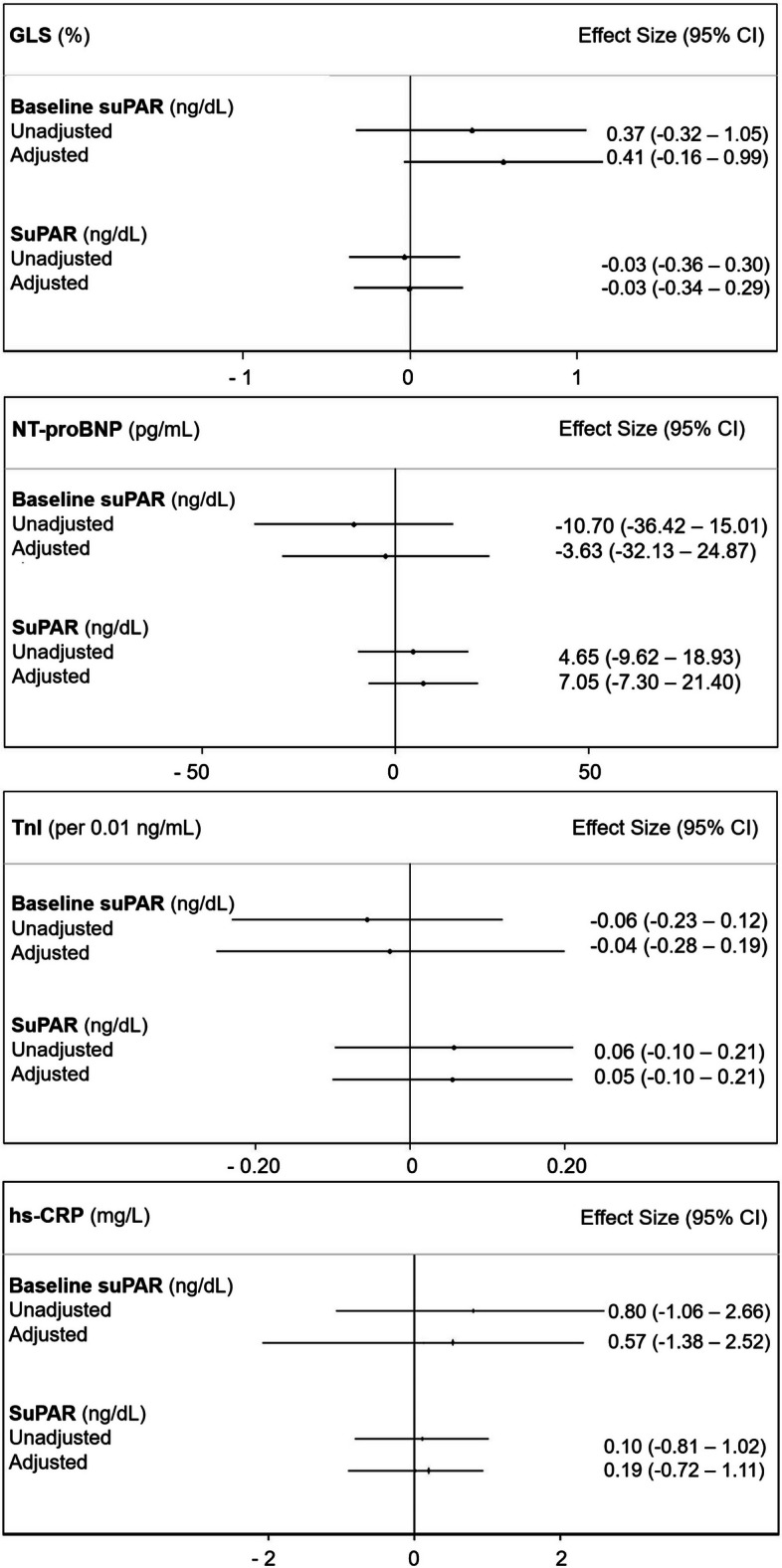
Estimates of association between baseline suPAR or serial suPAR measurements and the primary/secondary endpoints as markers of cardiotoxicity. Mixed effects linear regression was performed with adjustment for age, race/ethnicity, hypertension, dyslipidemia, diabetes, current smoker, body mass index, family history of premature ASCVD, aspirin, statin, ACE-i/ARB, and beta-blocker use

**Fig. 4:**
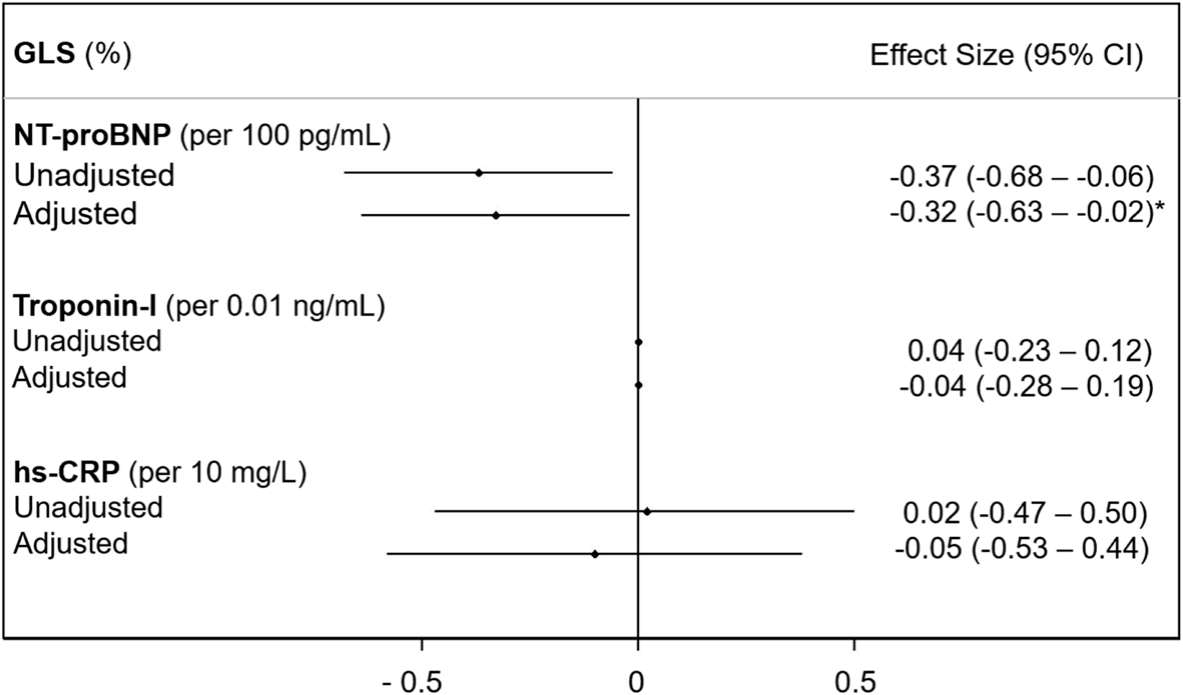
Estimates of association between GLS and cardiac biomarkers

There were no significant associations between suPAR and GLS or cardiac biomarkers in multivariable mixed effects models (all *p* > 0.05) (Fig. 3). Additionally, TnI and hs-CRP were not significantly associated with GLS (*p* > 0.05). NT-proBNP was negatively associated with GLS at borderline statistical significance (*p* = 0.04) (Fig. 4). About 5% of covariates were missing at random (Supplementary Table 2). The estimated associations did not change significantly after multiple imputations, although the association between NT-proBNP and GLS was no longer statistically significant (Supplementary Figure 1).

## Discussions

Cardiomyopathy is a known major complication of anthracycline treatment, particularly doxorubicin. Current methods for identifying cardiomyopathy rely on echocardiographic changes in LVEF, which lack sensitivity in detecting subclinical cardiotoxicity that precedes irreversible cardiomyopathy. Left ventricular GLS, NT-proBNP, TnI, and hs-CRP are well-studied markers of subclinical cardiotoxicity that may help to identify high-risk patients prior to clinically relevant disease. In this prospective study of breast cancer patients on standard-dose DOX, we did not find significant associations between suPAR and markers of subclinical cardiotoxicity. Of note, none of the study participants experienced clinically significant cardiomyopathy (based on LVEF) during treatment or over one-year follow-up. Furthermore, our study did not demonstrate any clinically meaningful changes in the primary or secondary endpoints (GLS or cardiac biomarkers) during DOX treatment or at follow-up.

The risk for DOX-induced cardiomyopathy is dose-dependent and studies have consistently shown a marked increase in incidence of cardiomyopathy in patients receiving cumulative doses greater than 300 mg/m^2^ [6, 30]. The follow-up time in our study includes the median time to onset of cardiomyopathy, which has been estimated to be 3.5 months following treatment [31]. It is likely that the lack of significant results and cardiotoxicity in our study is reflective of a baseline low-risk population along with a low risk of cardiotoxicity associated with the relatively low standard cumulative DOX dose of ≤ 240 mg/m^2^ in breast cancer patients [32]. The lack of significant cardiotoxicity at this lower cumulative DOX dose ≤ 240 mg/m^2^, is emphasized by the recent findings of the SUCCOUR and PRADA trials of subclinical cardiotoxicity and preventive medical therapy, respectively [33, 34]. In contrast, lymphoma, sarcoma, and leukemia patients are exposed to significantly higher cumulative doses of DOX – 300 mg/m2 up to maximum of 550 mg/m^2^ – and are therefore at relatively greater risk for cardiotoxicity.

The lack of cardiotoxicity in our study may also be a manifestation of the cohort’s favorable baseline cardiovascular profile compared with the general breast cancer population. The group was about 16 years younger than the median age of breast cancer diagnosis in the United States [35]. The younger age group was part of the inclusion/exclusion criteria for our study in order to capture a relatively healthy segment of the breast cancer population with less confounding factors relative to suPAR as a disease marker. The prevalence of ASCVD risk factors such as hypertension, diabetes mellitus, and dyslipidemia was comparable to estimates from contemporary breast cancer populations in the US – despite our cohort comprising of a higher proportion of black women who are known to have greater cardiovascular comorbidities at the time of diagnosis [36–38]. The baseline low-risk profile of our study population is also evidenced by relatively low baseline suPAR levels as a predictive marker of risk [23, 39]. The baseline suPAR levels in our cohort were similar to estimates from a large contemporary study of healthy, middle-aged American women (mean suPAR level of 2.62 ng/dL) – which further emphasizes the low risk profile of our study population [39]. Interestingly, one European study of suPAR in breast cancer patients found a median suPAR of 3.80 ng/dL. Although this was significantly higher than our estimates, it was derived from an older non-contemporary cohort that included patients with advanced stage disease who are expected to have a higher inflammatory state [23]. The relative paucity of suPAR studies of cancer cohorts underscores the need for further research to help determine clinically meaningful suPAR cutoff values.

The lack of cardiotoxicity is a major limitation to this study and reflects the low overall risk profile of our study population – as reflected by a young patient cohort with low (healthy) baseline suPAR levels who are exposed to lower cumulative DOX doses – as well as small sample size. Nonetheless, it appears to be an adequate sample size to conclude that the mean changes in the study endpoints (GLS and biomarker levels) over one-year follow-up were insignificant at a DOX dose ≤ 240 mg/m^2^. No participants experienced subclinical LV dysfunction based on GLS. As a result, our findings are unlikely to capture the true associations of suPAR and these cardiac markers across the full range of clinically observed endpoints. We also found a negative association between NT-proBNP and GLS of borderline statistical significance. This is most likely a spurious finding that further illustrates our study limitations, as prior studies have consistently established a positive association between NT-proBNP and GLS in various cardiac populations [40]. Even so, the prospective patient enrollment with objective lab data assessment is a strength of the study.

Despite these findings, suPAR remains a plausible marker of DOX-induced cardiotoxicity that merits further investigation. It is a well-studied inflammatory marker and risk factor for kidney disease that has been shown to be independently associated with clinically significant CVD and mortality [41]. A prior study found elevated levels of inflammatory biomarkers in breast cancer patients undergoing DOX chemotherapy as well as an association between increased inflammatory profile and decreased LVEF [42]. This suggests that patients who receive DOX may experience a continuous pro-inflammatory state that is linked to cardiac dysfunction. The inclusion of other promising inflammatory biomarkers, such as interleukin-6 and myeloperoxidase, should therefore be considered in the design of future investigations of DOX-induced cardiotoxicity. The lack of significant findings in our study is likely reflective of lower baseline risk of our study population and emphasizes the need for future studies that include a wider range of baseline suPAR levels in a larger sample size of lymphoma, sarcoma, and leukemia patients who are exposed to higher cumulative DOX doses and are therefore more susceptible to clinically evident cardiotoxicity. Finally, our findings represent a single-center experience with a unique patient demographic; future, prospective multi-center studies will improve generalizability.

## Conclusions

In our study, standard DOX-based chemotherapy for breast cancer at a cumulative dose of 240 mg/m^2^ was associated with an overall low risk cardiotoxicity profile. In this study cohort with baseline lower suPAR levels reflective of baseline lower risk profile, suPAR was not associated with other markers of subclinical cardiotoxicity. Future studies of suPAR and DOX-induced cardiotoxicity should include a larger sample size in leukemia and lymphoma patients, who traditionally receive higher cumulative doses (≥ 300 mg/m^2^) of DOX; comparing lower to higher baseline suPAR levels as markers of baseline risk.

### Clinical perspective

- In this prospective cohort study of breast cancer patients receiving standard-dose doxorubicin, there was no evidence of subclinical or overt doxorubicin-induced cardiotoxicity based on global longitudinal strain imaging and cardiac biomarkers.
- Across this narrow range of clinical endpoints, soluble urokinase plasminogen activator receptor was not associated with any of these markers of cardiotoxicity.
- Further research is needed to clarify the associations between soluble urokinase plasminogen activator receptor and markers of cardiotoxicity across a broader range of clinical endpoints and should therefore include leukemia, lymphoma, and sarcoma patients who are more likely to experience therapy-related cardiotoxicity.

### Supplementary Information

**Additional file 1: Supplementary Table 1.** Baseline demographic variables of patients who withdrew participation from the study. **Supplementary Table 2.** Tabulation of missing variables over study follow-up over 222 total observations during follow-up. **Supplementary Figure 1.** Changes in cohort measurements of SuPAR, GLS, NT-proBNP, TnI, and hs-CRP over study follow-up. **Supplementary Figure 2.** Estimates of association between baseline suPAR or serial suPAR measurements and the primary/secondary endpoints as markers of cardiotoxicity using multiply imputed datasets. Mixed effects linear regression was performed with adjustment for age, race/ethnicity, hypertension, dyslipidemia, diabetes, current smoker, body mass index, family history of premature ASCVD, aspirin, statin, ACE-i/ARB, beta-blocker.

## Data Availability

The datasets generated and/or analysed during the current study are not publicly available but are available from the corresponding author on reasonable request.

## Abbreviations

ASCVD: Atherosclerotic cardiovascular disease
CABG: Coronary artery bypass graft
DOX: Doxorubicin
ESRD: End stage renal disease
GLS: Global longitudinal strain
hs-CRP: High-sensitivity C-reactive protein
LVEF: Left ventricular ejection fraction
NT-proBNP: N-terminal prohormone B-type natriuretic peptide
PCI: Percutaneous coronary intervention
SuPAR: Soluble urokinase plasminogen activator receptor
TnI: Troponin-I
