# Correction: Soluble urokinase plasminogen activator receptor and cardiotoxicity in doxorubicin‑treated breast cancer patients: a prospective exploratory study

**DOI:** 10.1186/s40959-024-00205-5

**Published:** 2024-02-06

**Authors:** Jian Chu, Lillian Tung, Issam Atallah, Changli Wei, Melody Cobleigh, Ruta Rao, Steven B. Feinstein, Lydia Usha, Kathrin Banach, Jochen Reiser, Tochukwu M. Okwuosa

**Affiliations:** 1 Department of Internal Medicine, Rush University Medical Center, Chicago, IL USA; 2 Department of Internal Medicine, Division of Cardiology, Saint Louis University Hospital, St. Louis, MO USA; 3 Department of Internal Medicine, Section of Hematology/Oncology, Rush University Medical Center, Chicago, IL USA; 4 Department of Internal Medicine, Division of Cardiology, Rush University Medical Center, 1717 West Congress Parkway | Kellogg Bldg, Suite 328, Chicago, IL 60612 USA

**Correction: Cardio-Oncology 10, 3 (2024)**

10.1186/s40959-023-00191-0

Following publication of the original article [1], the authors identified an error in the author name of Issam Atallah.

The incorrect author name is: Issam Attalah.

The correct author name is: Issam Atallah.

The author group has been updated above and the original article [1] has been corrected.
