# Supplementary material for: Soluble urokinase plasminogen activator receptor and cardiotoxicity in doxorubicin-treated breast cancer patients: a prospective exploratory study
Source: Cardiooncology. 2024 Jan 15;10:3. doi: 10.1186/s40959-023-00191-0 (PMC10788987; doi:10.1186/s40959-023-00191-0)
Supplement: Supplementary file 1 — Additional file 1: Supplementary Table 1. Baseline demographic variables of patients who withdrew participation from the study. Supplementary Table 2. Tabulation of missing variables over study follow-up over 222 total observations during follow-up. Supplementary Figure 1. Changes in cohort measurements of SuPAR, GLS, NT-proBNP, TnI, and hs-CRP over study follow-up. Supplementary Figure 2. Estimates of association between baseline suPAR or serial suPAR measurements and the primary/secondary endpoints as markers of cardiotoxicity using multiply imputed datasets. Mixed effects linear regression was performed with adjustment for age, race/ethnicity, hypertension, dyslipidemia, diabetes, current smoker, body mass index, family history of premature ASCVD, aspirin, statin, ACE-i/ARB, beta-blocker. [file 40959_2023_191_MOESM1_ESM.docx]

**Supplementary Materials**

**Supplementary Table 1.** Baseline demographic variables of patients who withdrew participation from the study

| **Variables** |  | N = 5 |
| --- | --- | --- |
| **Age (yrs)** |  | 50.8 ± 5.4 |
| **White** |  | 0 (0%) |
| **ASCVD Risk Factors** | **Hypertension** | 1 (20%) |
|  | **Dyslipidemia** | 0 (0%) |
|  | **Diabetes** | 1 (20%) |
|  | **Family History** | 2 (40%) |
|  | **Current Smoker** | 0 (0%) |
|  | **Body Mass Index (kg)** | 33.9 ± 8.3 |
|  | **Chronic Kidney Disease** | 0 (0%) |
| **Cardiac Medications** | **ACE-i/ARB** | 1 (20%) |
|  | **Beta-blocker** | 0 (0%) |
|  | **Statin** | 0 (0%) |
|  | **Aspirin** | 0 (0%) |
| **Breast Cancer Characteristics** | **Left-sided** | 4 (80%) |
|  | **Stage I** | 0 (0%) |
|  | **Stage II** | 2 (40%) |
|  | **Stage III** | 3 (60%) |
|  | **ER+** | 2 (40%) |
|  | **PR+** | 1 (20%) |
| **Treatment Characteristics** | **DOX dose (mg/m^2^)** | 240.0 ± 0.0 |
|  | **Cyclophosphamide Use** | 5 (100%) |
|  | **Paclitaxel Use** | 5 (100%) |
|  | **Adjuvant radiation** | 5 (100%) |
| **Cardiac Biomarkers** | **NT-proBNP (pg/mL)** | 43.6 (15.6, 74.6) |
|  | **TnI (ng/dL)** | 0.01 (0.01, 0.01) |
|  | **hs-CRP (mg/L)** | 10.1 (1.4, 13.5) |

**ACE-i** = angiotensin-converting enzyme inhibitor; **ARB** = angiotensin receptor blocker; **DOX** = doxorubicin; **ER** = estrogen receptor; **hs-CRP** = high-sensitivity C-reactive protein; **NT-proBNP** = N-terminal pro B-type natriuretic peptide; **PR** = progesterone receptor; **TnI** = conventional troponin-I

**Supplementary Table 2.** Tabulation of missing variables over study follow-up over 222 total observations during follow-up.

| **Variable** | **Missing (%)** |
| --- | --- |
| SuPAR | 14 (6.3%) |
| GLS | 13 (5.9%) |
| NT-proBNP | 10 (4.5%) |
| TnI | 10 (4.5%) |
| hs-CRP | 14 (6.3%) |


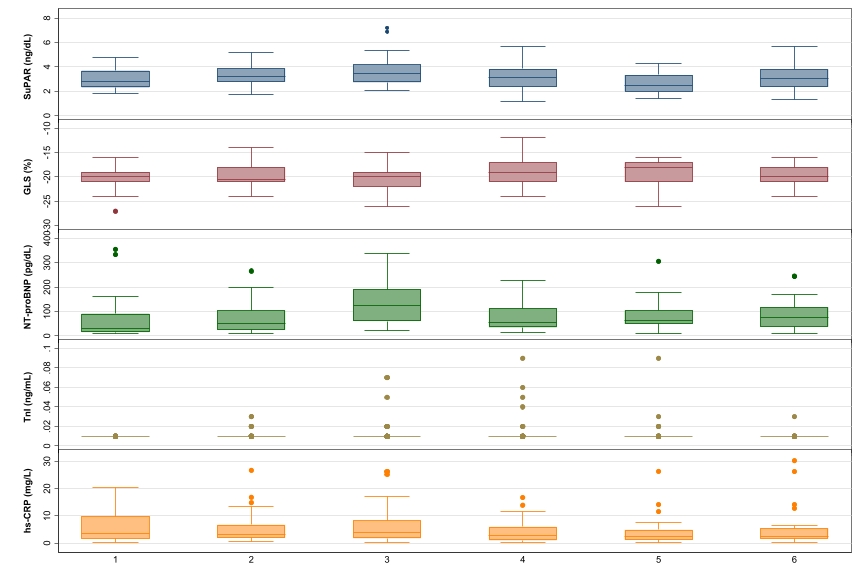


**Supplementary Figure 1.** Changes in cohort measurements of SuPAR, GLS, NT-proBNP, TnI, and hs-CRP over study follow-up.

**
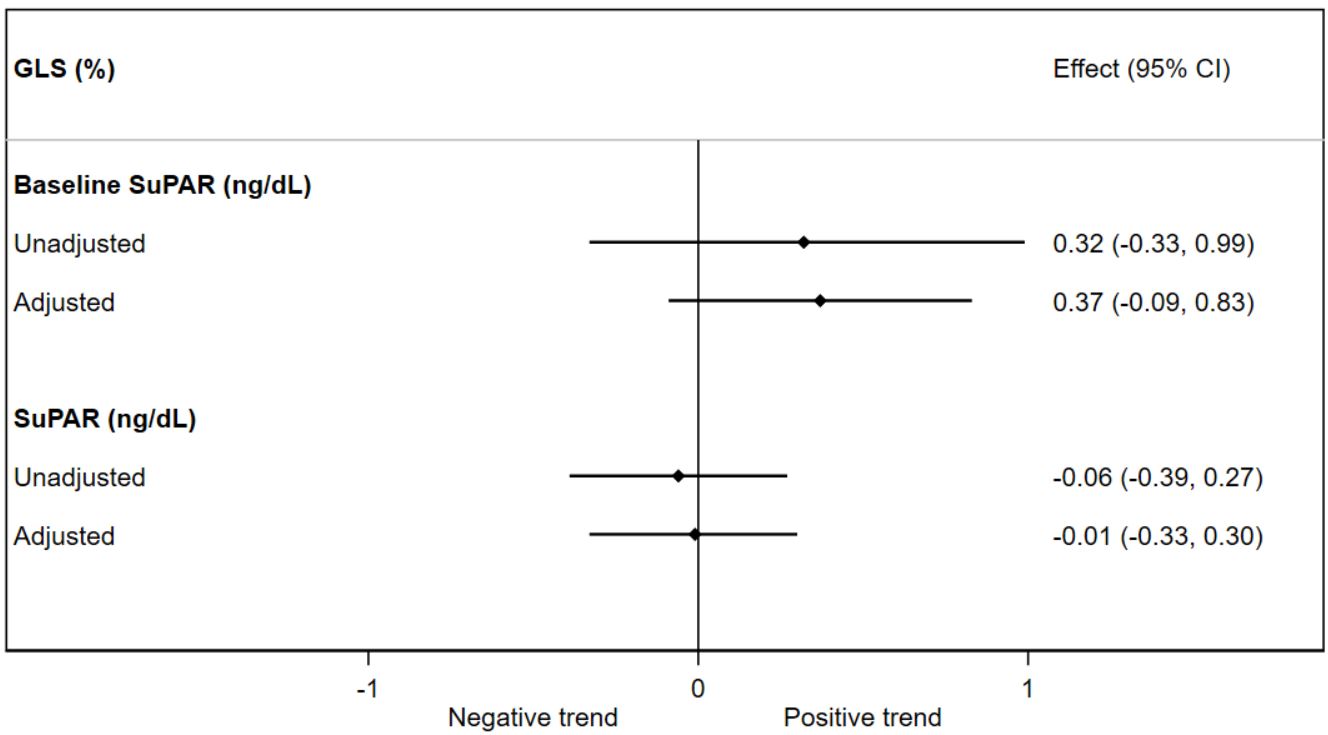

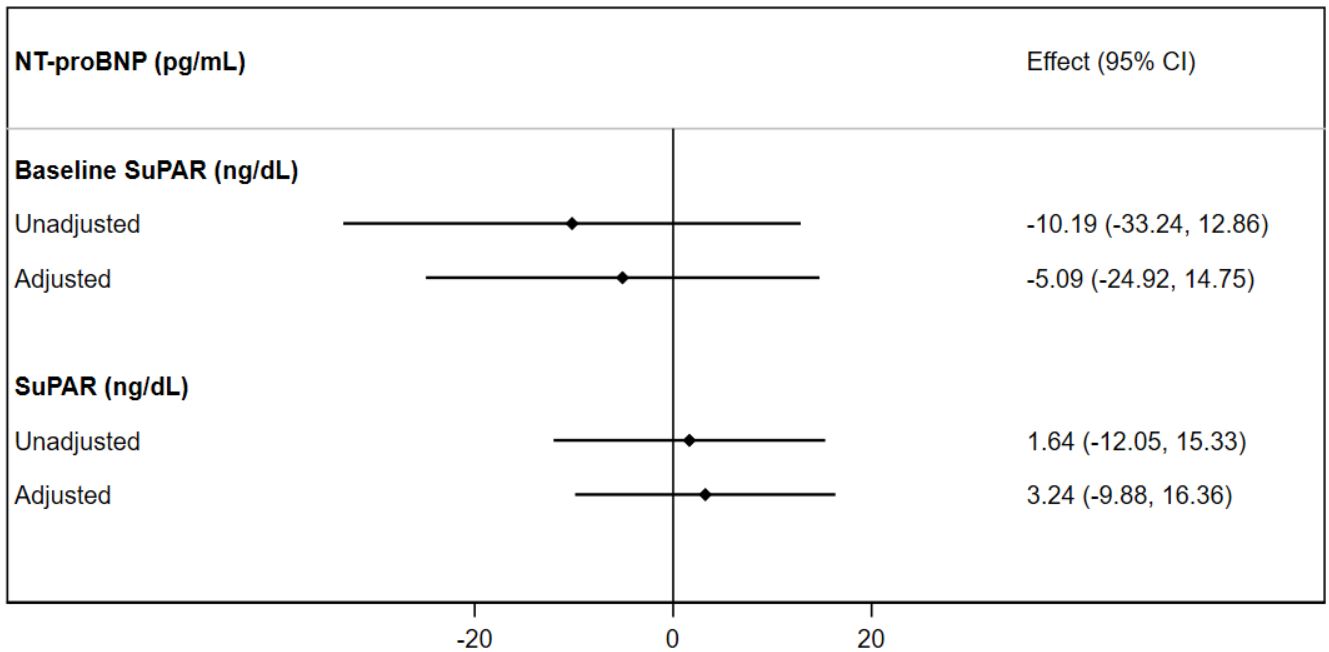

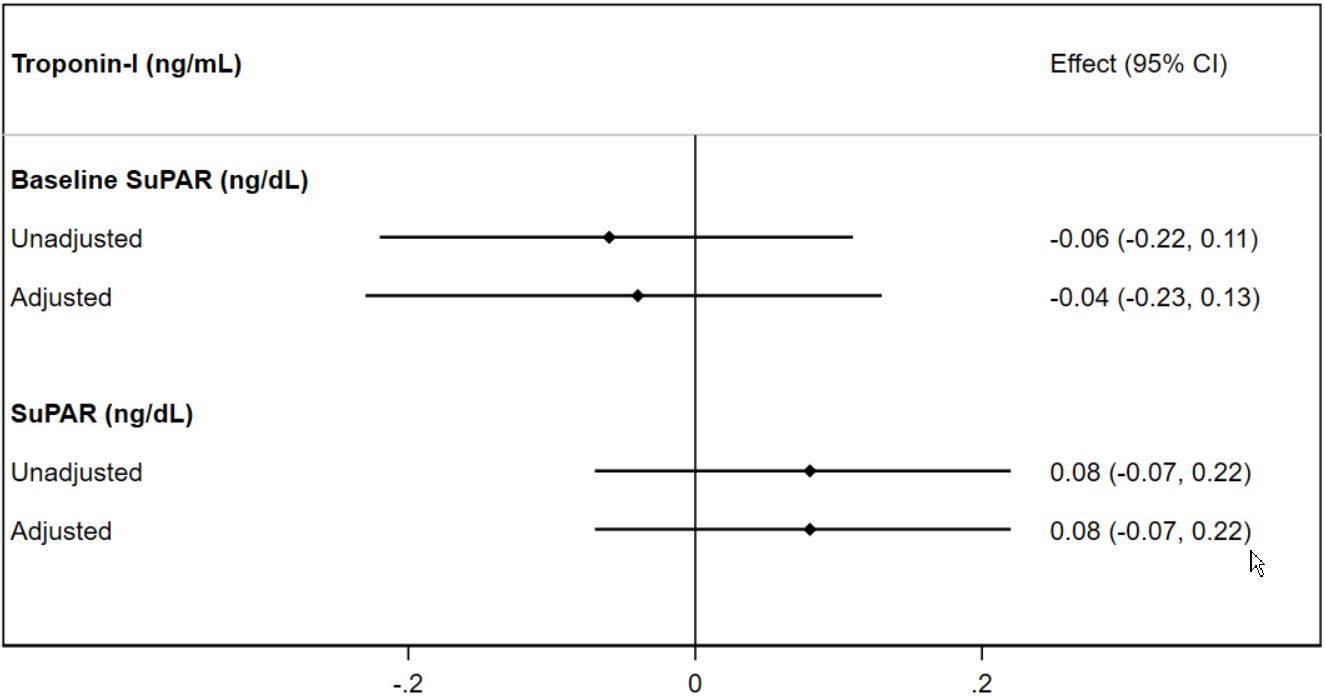

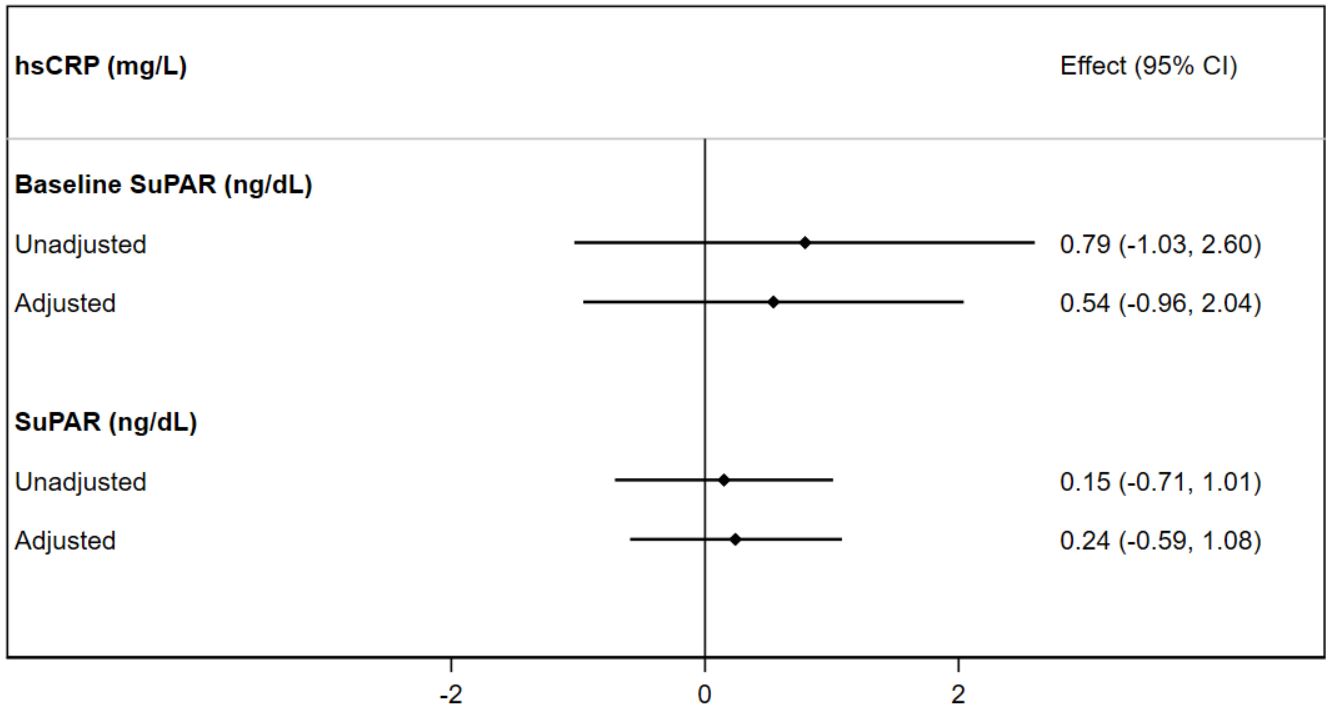

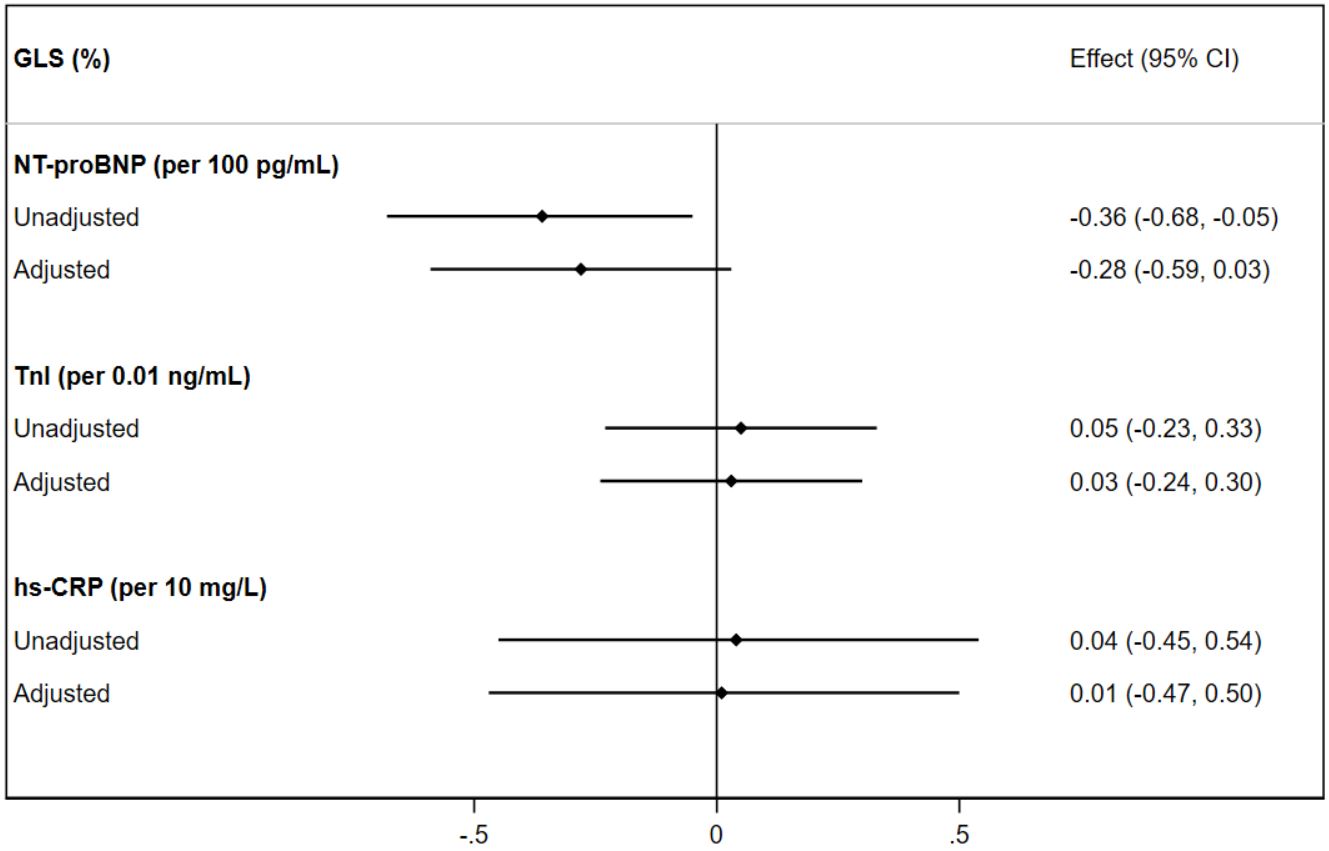
**

**Supplementary Figure 2.** Estimates of association between baseline suPAR or serial suPAR measurements and the primary/secondary endpoints as markers of cardiotoxicity using multiply imputed datasets. Mixed effects linear regression was performed with adjustment for age, race/ethnicity, hypertension, dyslipidemia, diabetes, current smoker, body mass index, family history of premature ASCVD, aspirin, statin, ACE-i/ARB, beta-blocker.
